# *Trypanosoma cruzi* DTU parasite diversity and clinical outcomes in mesoregions of the Northeast Brazilian State of Pernambuco

**DOI:** 10.1371/journal.pntd.0013996

**Published:** 2026-02-13

**Authors:** Thayse do Espírito Santo Protásio da Silva, Lucia Elena Alvarado-Arnez, Angelica Martins Batista, Silvia Marinho Martins Alves, Gloria Melo, Cristina Veloso Carrazzone, Wilson Oliveira Jr, Constança Britto, Samanta Cristina das Chagas Xavier, Otacilio C. Moreira, Joseli Lannes-Vieira

**Affiliations:** 1 Laboratório de Biologia das Interações, IOC/Fiocruz, Rio de Janeiro, Brazil; 2 Laboratório de Hanseníase, IOC/Fiocruz, Rio de Janeiro, Brazil; 3 Ambulatório de Doença de Chagas e Insuficiência Cardíaca, Pronto Socorro Cardiológico de Pernambuco (PROCAPE)/Universidade de Pernambuco, Recife, Brazil; 4 Laboratório de Biologia Molecular e Doenças Endêmicas, IOC/Fiocruz, Rio de Janeiro, Brazil; 5 Laboratório de Biologia de Tripanosomatídeos, IOC/Fiocruz, Rio de Janeiro, Brazil; 6 Laboratório de Virologia e Parasitologia Molecular, IOC/Fiocruz, Rio de Janeiro, Brazil; Instituto de Investigaciones Biotecnológicas, ARGENTINA

## Abstract

**Background:**

Chagas disease (CD), caused by infection with the protozoan parasite *Trypanosoma cruzi*, is a neglected tropical illness that affects 6–7 million individuals worldwide. CD may progress to chronic cardiac (CARD), digestive (DIG), or cardio-digestive (CARD/DIG) forms. Parasite genetic diversity, defined in discrete typing units (DTUs) TcI-TcVI and TcBat, may contribute to clinical forms. Here, we challenged this idea by studying a population born in the State of Pernambuco, Northeast of Brazil.

**Methodology/Principal Findings:**

Patients born in eco-geographically distinct mesoregions (Sertão, Agreste, Zona da Mata, and Metropolitan Region) attending the referral PROCAPE hospital were serologically diagnosed for CD and characterized as non-CARD (16.5%), CARD (67%), DIG (12.1%), and CARD/DIG (4.3%). Out of 346 CD patients not subjected to etiological treatment, 128 (37%) were positive for conventional PCR targeting *T. cruzi* kDNA. DTU genotyping using pre-established primers and algorithms revealed 85/128 (66%) samples amplified for at least one target, and of these, 49 (58%) were classified into DTUs, showing a higher distribution of DTUs TcV (16) and TcIII (13) but also TcIV (7), TcI (3), TcVI (3), and TcII (1). Mixed infections by TcI + TcV (2), TcIII + TcIV (3), and TcIII + TcV (1) were also found. Out of the 49 classified samples, 36 were CARD patients (73.5%), infected mainly by TcV (13) and TcIII (10). Considering patients’ birthplace, in the Agreste, all DTUs were detected, with prevailing TcV and TcIII; in Zona da Mata, DTU characterization reflected the input of samples (21/49, 43%), again mainly TcV and TcIII.

**Conclusion/Significance:**

DTUs TcI-TcVI were only detected in the blood of patients born in the Agreste, area of ecological transition. Although not frequently found in human infections in Brazil, the prevailing TcV, TcIII, and TcIV were detected in patients born in Pernambuco. These data could not disclose an identifiable association of *T. cruzi* DTU circulating in the blood of patients with clinical forms of CD.

## Author summary

Chagas disease (CD), a neglected disease that affects 6-7 million individuals worldwide, may have different clinical manifestations such as CARD, CARD/DIG, and DIG forms. The etiological agent of CD, the flagellate protozoan parasite Trypanosoma cruzi shows a significant biological and genetic diversity. T. cruzi, isolates are classified and grouped by genetic similarity and common molecular markers into seven discrete typing units (DTUs) – TcI to TcVI, and TcBat. To date, there is insufficient evidence to associate the use of T. cruzi genotypes as biomarkers for clinical stage outcome and/or disease progression. However, TcI has been associated with the CARD form in some populations. Here, in a group of clinically classified Brazilian chronic CD patients, we genotyped the T. cruzi parasite in DTUs and explored its possible association with the different clinical forms. These data support the lack of association between T. cruzi genotype and clinical forms of CD. However, we bring new evidence showing a predominance of TcV and TcIII DTUs in chronic CD patients born in Pernambuco, Northeastern Brazil, indicating an underreporting of the geographic distribution of T. cruzi DTUs circulating in Brazil.

## Introduction

Chagas disease (CD) is an anthropozoonosis and parasitic infection caused by the flagellated protozoan parasite *Trypanosoma cruzi*, recognized for over a century and affecting mainly neglected populations. It is estimated that around 6–7 million individuals are infected worldwide, and 1.9 to 4.2 million of them are Brazilians with chronic CD, requiring treatment and medical monitoring [1,2]. The disease is characterized by having two distinct phases: the acute phase, which is usually asymptomatic, and the chronic phase, which can lead to different outcomes, ranging from the indeterminate form, with a benign prognosis, to the cardiac (CARD), digestive (DIG) or cardio-digestive (CARD/DIG) forms [1,3]. CD is a multifactorial disease, and the clinical forms and severity may be related to host/parasite interplay, which may involve the parasite infecting strain, route of transmission, and host traits such as genetic, previous immune status and the presence of comorbidities [3–5]. Pre-clinical studies suggest that host and parasite factors directly influence the clinical manifestations of CD [6].

Based on an expert consensus, *T. cruzi* isolates were classified and grouped by genetic similarity and common molecular markers into six discrete typing units (DTUs) – TcI to TcVI, and a seventh isolate initially described in bats, TcBat [7,8]. Nevertheless, all these lineages of *T. cruzi* can infect humans [9,10]. The *T. cruzi* parasite has great genetic diversity and different geographic distribution; therefore, typing methods allow us to challenge possible associations between the parasite and eco-epidemiological characteristics, transmission cycles, and clinical manifestations of CD [9,11,12]. To date, there is insufficient evidence to associate the use of *T. cruzi* genotypes as a biomarker for stage and/or clinical progression, despite TcI being associated with the CARD form of CD in Colombia and Venezuela [13]. The TcII, TcV, and TcVI DTUs have been related to all clinical manifestations of the disease [14–16]. On the other hand, TcIII and TcIV DTUs are more frequently associated with the sylvatic cycle and oral outbreaks in the Amazon region [13,17,18]. Although TcIII DTU has a wide distribution in triatomines and mammal reservoirs, it has been also found in humans in some Brazilian States (Rio Grande do Norte, Paraná, Mato Grosso do Sul, Rio de Janeiro, Pará, Bahia and Goiás) [19–21]. Indeed, this genotype was identified in an outbreak of oral CD in a rural area of the state of Amazonas in 2007 [22]. Later, TcIII DTU was detected in patients with the chronic indeterminate form in the Northeastern state of Brazil, Rio Grande do Norte [23]. Also, TcIV DTU appears to be present in patients from Colombia and Venezuela [24,25]. There are few studies on the genetic diversity of *T. cruzi* parasite in Brazilian patients, with a focus on chronic patients and frequently using biological filters as isolates after hemoculture [9,12,23,26].

Parasite DTUs exhibit a diversity of pathogen-associated molecular patterns, which explains the antigenic variability of different strains of *T. cruzi* and influences the host immune response, potentially interfering with clinical outcomes and responses to therapeutic interventions [10]. The drug of the first choice for treating the acute phase of CD is benznidazole (Bz), which has a cure rate of 50–80% of cases [27]. The primary objective of etiological treatment for CD is to achieve parasitological cure, aiming to reduce the risk of developing symptomatic forms and prevent the transmission of the parasite [28]. Several studies have shown that different DTUs of *T. cruzi* may exhibit differential sensitivity to trypanocidal drugs, and treatment may potentially mask the presence of mixed infections in genotyping studies. Therefore, it is essential to conduct work that takes this possibility into account and to avoid the use of blood culture, which can also select more resistant strains [13,29].

The Brazilian territory is extensive and presents diverse ecosystems with incredible biodiversity, particularly concerning vectors and wild mammalian hosts of *T. cruzi* [30]. Therefore, it is expected that the natural history of CD has regional particularities. In the present study, we focused on the State of Pernambuco, located in the Northeast of Brazil. This state has a total area of approximately 98,067.879 km² and a population of 9,058,931 inhabitants in 2022, with an estimated 9,562,007 inhabitants by July 2025 [31,32]. Further, the State of Pernambuco is administratively divided into mesoregions based on geographic, predominant climate, vegetation, and socioeconomic characteristics. The Sertão mesoregion, currently encompassing Sertão and Sertão do São Francisco [31], has a semi-arid climate with a predominance of Caatinga vegetation [31]. The Agreste mesoregion has a semi-arid climate like that of the Sertão and a humid climate near the coast. The characteristic biome of this region is a transition from the Caatinga to the Atlantic Forest [31]. The humid climate and predominance of the Atlantic Forest biome characterize Zona da Mata. The Metropolitan Region has a tropical and humid climate, characterized by the predominance of the Atlantic Forest biome [31]. In 1980, the first national serological survey estimated the prevalence of CD cases as 4.22% in Brazil and 2.8% in the state of Pernambuco [33]. Recently, a study monitoring chronic CD from 2019 to 2024 in the state of Pernambuco revealed that out of 8,873 people who underwent serological testing for CD, 1,162 were considered reactive for IgG antibodies to *T. cruzi*, which corresponds to a positivity rate of 13.10%, with 56% being women and 74% over 50 years of age. Furthermore, a mortality rate of 6.65/100,000 inhabitants was observed, with a case fatality rate of 50.9% for chronic CD [34]. Although these studies use different tools, target populations, different objectives, and are separated by a significant time interval, they corroborate the persistent epidemiological relevance of CD in the State of Pernambuco.

To contribute to the epidemiological knowledge of *T. cruzi* DTUs distribution in Brazil, we investigated the possible influence of the parasite diversity on the different clinical forms of chronic CD in a well-defined study group in terms of clinical parameters and place of birth, reaching different mesoregions of the State of Pernambuco/Brazil. Molecular diagnosis and *T. cruzi* genotyping were evaluated directly from the blood samples of patients not treated with Bz and clinically classified as non-cardiac (non-CARD), CARD (mild – B1; moderate – B2; and severe – C), digestive (DIG) and cardio-digestive (CAR/DIG) forms [35,36].

## Patients and methods

### Ethical statement

This study was conducted in accordance with the recommendations of the Ethics Committees of Oswaldo Cruz Foundation – Fiocruz/RJ (541/09) and Pronto Socorro Cardiológico de Pernambuco” of the University of Pernambuco – PROCAPE/UPE (80210/10). All participants provided written informed consent in accordance with the principles outlined in the Declaration of Helsinki.

### Study population

A group of 346 patients born and resident in the State of Pernambuco, Northeast of Brazil, was enrolled in a cross-sectional observational study conducted over 5-years (2010–2015) at the “Ambulatório de Doença de Chagas e Insuficiência Cardíaca do Pronto Socorro Cardiológico de Pernambuco” of the University of Pernambuco (PROCAPE/UPE), located in the City of Recife, Pernambuco. At enrollment, 10 mL of peripheral blood was collected to confirm the serological diagnosis and DNA isolation. Following the recommendation of the Brazilian Consensus on CD [35], the serological diagnosis was determined by two independent tests, that included an enzyme-linked immunosorbent assay (ELISA) and an indirect immunofluorescence test, performed by the Central Reference Laboratory from Pernambuco (LACEN-PE, Brazil). Serological results were added to patients’ medical records. Patients under 18 years of age, those diagnosed with co-infections, alcohol users, vulnerable populations (indigenous people, Quilombola, pregnant women, people with chronic mental illnesses), or who had undergone previous treatment with specific therapy for CD were not included in the present study.

### Study design

After selecting patients with CD seropositive results released from LACEN-PE, and who had not been treated with trypanocidal drugs, we carried out molecular analyses as a single-blind study. After molecular diagnosis by conventional polymerase chain reaction (PCR) targeting *T. cruzi* kDNA, positive DNA samples were used for genotyping *T. cruzi* DTUs, using pre-established primers and algorithms. For global analyses, the study population was characterized according to clinical and demographic parameters, and all data were obtained from the patients’ medical records and the applied questionnaire. The classification of the chronic phase of CD regarding the presence or absence of cardiac form was carried out according to the I Latin American Guideline for Diagnosis and Treatment of Chagasic Cardiopathy [36]. According to the evaluated criteria, patients were classified as follows: stage A (non-CARD), without symptoms of digestive and heart disease, and with normal electrocardiographic (ECG) and echocardiographic (ECHO) recordings; stage B1 (mild CARD), patients without clinical signs of heart failure (HF), ECG or ECHO with segmental dysfunction, but with normal ventricular function; stage B2, patients with global ventricular dysfunction with decreased left ventricular ejection fraction (LVEF); and stage C (severe CARD), patients with clinical signs of HF, ECG changes and structural cardiomyopathy by ECHO assessment, including reduction in LVEF (< 45%; Simpson’s method). Regarding the digestive form, the radiological characteristics were analyzed as suggested in the Brazilian Consensus on Chagas Disease [37], and the classification was performed into groups I to IV according to the severity of the DIG form to allow clinical follow-up; however, due to the reduced number of DIG patients in these groups, they were referred to as DIG. When possible, obtained findings were geographically distributed according to mesoregions, using the map and the List of Municipalities of Pernambuco [31,38].

### Blood collection and transport

10 mL of peripheral blood was collected from each patient intravenously, 5 mL in a tube containing EDTA as anticoagulant (Code 367861, BD Vacutainer) and 5 mL in a dry tube (Code 367812, BD Vacutainer) to obtain serum. The biological material was stored at −70°C at the “Laboratório de Células Tronco” (PROCAPE/UPE) and subsequently sent to the “Laboratório de Biologia das Interações”, at the Oswaldo Cruz Institute/Fiocruz/RJ (LBI/Fiocruz), where it was properly stored. Sample transport was carried out in accordance with the International Air Transport Association (IATA) standards. All patients donated blood only once, as recommended by the Ethics Committees of PROCAPE/UPE.

### *T*. *cruzi* reference samples

Epimastigote forms of *T*. *cruzi* strains Dm28c (TcI), Y (TcII), INPA 3663 (TcIII), INPA 4167 (TcIV), LL014 (TcV), and CL (TcVI) were obtained from the “Coleção de Protozoários da Fundação Oswaldo Cruz”. Parasites were cultivated in brain-heart infusion (BHI) medium, supplemented with 16 mg/mL haemin and 10% (v/v) heat-inactivated fetal bovine serum, at 28°C. At the logarithmic phase of growth, cells were harvested by centrifugation and washed three times in phosphate-buffered saline (PBS) pH 7.4 before use.

### DNA isolation from clinical samples and *T*. *cruzi* reference strains

To avoid contamination, genomic DNA from clinical samples was isolated at the LBI/Fiocruz, and the genomic DNA from reference samples was obtained at the Laboratório de Biologia Molecular e Doenças Endêmicas, IOC/Fiocruz. After thawing, blood samples were mixed with 6M guanidine/0.2M EDTA pH 8.0 at 1:1 proportion to produce the Guanidine-EDTA blood (GEB) lysate. Subsequently, 1 mL GEB samples were boiled for 1 minute to cleave the kDNA network [39] and left at room temperature overnight. The isolation of genomic DNA was carried out from 300 μL GEB using the commercial kit “High Pure PCR Template Preparation Kit” (Roche Diagnostics Corp., Indianapolis, IN, USA. Cat. No. 11796828001), as previously reported [40]. In the final step of the protocol, DNA was eluted in 100 µL of elution buffer and stored at −20 °C until use. One negative extraction control (seronegative blood for CD) was included in each DNA extraction batch, together with 11 GEB samples. To isolate DNA from standard strains of *T. cruzi* DTUs, blood samples from healthy donors (negative *T. cruzi* serology) resident in Rio de Janeiro were spiked with epimastigote forms of the parasite, and DNA isolation was carried out as described above.

### Molecular diagnosis by conventional PCR

Conventional PCR assays were carried out in a final volume of 50 μl, containing: 5 μl DNA, 5 μl 10 × Taq Platinum buffer, 0.2 mM dNTPs, 4.5 mM MgCl_2_, 1.25 U Taq Platinum DNA polymerase (Life Technologies, Carlsbad, CA, USA), 200 nM 121/122 primers (*T. cruzi* kDNA) [39], as described in **S1 Table**. Amplifications were performed in the GeneAmp PCR System 9700 (Life Technologies), as described in **S2 Table**. In parallel, the DNA samples that presented negative results for the *T. cruzi* kDNA target were subjected to a second PCR assay, under the same cycling conditions, using oligonucleotides for the human β-globin gene (200 nM) PCO3 and PCO4 (**S1 Table**), as previously described [39,41]. PCR products were applied to 2% (w/v) agarose gels and submitted to electrophoresis at 80 V for 40 min. Gels were stained with GelRed (Biotium). Controls were added to all assays: No Template Control as well as DNA extraction negative controls and positive controls (samples artificially spiked with *T cruzi* strains: CL Brener, TcVI DTU or Y, TcII DTU). The samples were considered positive for *T. cruzi* kDNA or the β-globin gene when fragments of approximately 330 bp or 110 bp, respectively, were identified.

### Molecular genotyping of *T. cruzi*

**Fig 1:**
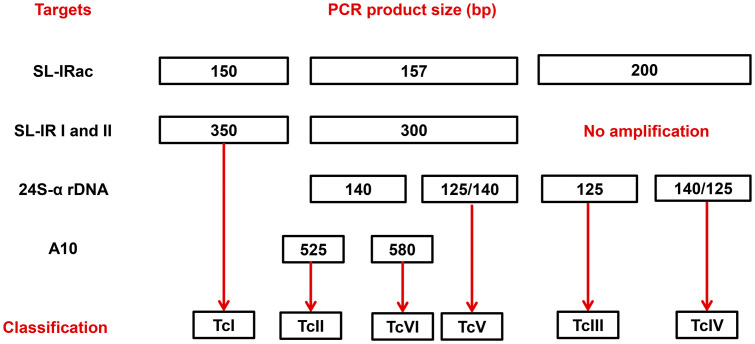
Flowchart used for classification of *Trypanosoma cruzi* DTUs. Algorithmic showing the PCRs targets and PCR products size (bp). The intergenic region of Spliced Leader (SL-IRac) [UTCC and TCac primers] was used to distinguish between TcI (150 bp), TcII, V or VI (157 bp) and TcIII or TcIV (200 bp); SL-IR I and II [TCC, TC1 and TC2 primers], to distinguish between TcI (350 bp), TcII, TcV and TcVI (300 bp) and TcIII and TcIV (not amplified)]; D7 domain of the 24Sα ribosomal RNA gene [Heminested PCR: D75 and D76 (first round) and D76 and D71 (second round) were used to distinguish between TcII and TcVI (140 bp), TcIII (125 bp), TcIV (140/145 bp) and TcV (125 or 125 + 140 bp)]; A10 nuclear fragment [Heminested PCR: Pr1 and P6 (first round) and Pr1 and Pr3 (second round), to differentiate TcII (690/580 bp) from TcVI (630/525 bp)].

*T*. *cruzi* genotyping into DTUs from I to VI was performed based on conventional multilocus PCR and an algorithm with clear decision points, as reported [29]. As a panel of positive controls, we used *T*. *cruzi* epimastigotes from subpopulations classified as DTUs TcI to TcVI (clones/strains: Dm28c (TcI), Y (TcII), INPA 3663 (TcIII), INPA 4167 (TcIV), LL014 (TcV), and CL-Brener (TcVI), obtained from the Protozoan Collection of the Oswaldo Cruz Foundation (Colprot-IOC/Fiocruz). Primer sequences and thermocycling steps are described in **S1 Table**. The amplification assays were performed as described in **S2 Table**, using 5 μL of extracted DNA of the kDNA-positive samples. The genotyping sensitivity was up to 0.5 par. Eq./mL for all targets analyzed [12]. PCR products (25 μL) were separated by agarose gel electrophoresis (3% for SL IRac; 2% for SL-IR I and II; 3% for 24Sα rDNA and 2% for A10, 80V, 1 hour) and stained with Gel Red (Biotium) 0.1 X and visualized under UV light. **Fig 1** shows the algorithmic used for classification of *Trypanosoma cruzi* DTUs.

### Geospatial analysis

For mapping the distribution and visualization of the genetic diversity of *T. cruzi* and clinical forms of Chagas disease in the study area, a Geographic Information System (GIS) was utilized for spatial analysis and thematic map construction in Quantum GIS software QGIS 3.34 ‘Prizren’ version (https://www.qgis.org). Spatial data were obtained from the Brazilian Institute of Geography and Statistics (IBGE), using the Digital Municipal Grid (Malha Municipal Digital—MMD), available under a Creative Commons Attribution 4.0 International license [42] https://www.ibge.gov.br/geociencias/organizacao-do-territorio/malhas-territoriais/15774-malhas.html.

### Statistical analysis

When appropriate, data were analyzed, and arithmetic means and standard deviations were established, or medians were calculated. Statistical tests, such as chi-square, Fisher’s, and ANOVA with Bonferroni post-test, Cochran-Armitage trend-test, were performed using PRISMA 8.0 software and R packages (epiDisplay, CATT). Differences were considered significant when *p* < 0.05.

## Results

### Epidemiological characteristics of the study group

**Fig 2:**
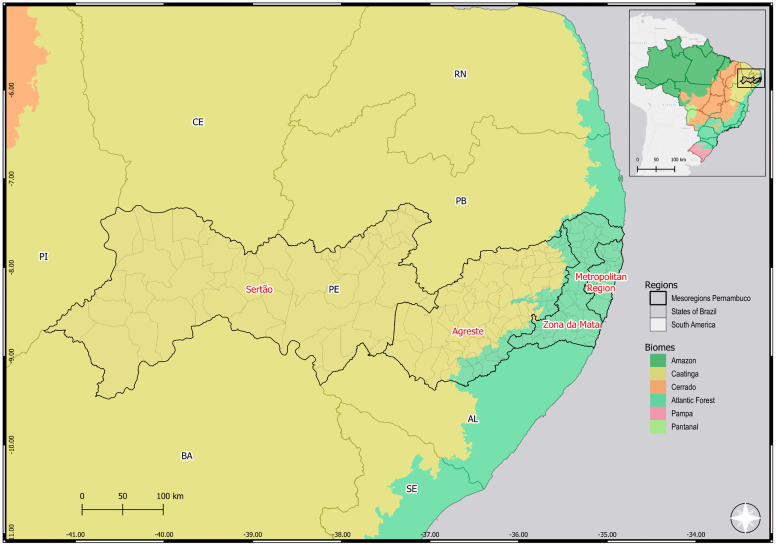
Maps showing the geographical location of the different mesoregions of Pernambuco/Brazil. Map showing the location of the state of Pernambuco on maps of Brazil and South America, and the location of the different mesoregions. This map was created using QGIS 3.34 ‘Prizren’ version software and cartographic bases obtained from the Brazilian Institute of Geography and Statistics (https://ibge.gov.br/). Source: Brazilian Institute of Geography and Statistics (IBGE). Digital Municipal Grid (MMD). Licensed under CC BY 4.0. Municipality border shape available from https://www.ibge.gov.br/geociencias/organizacao-do-territorio/malhas-territoriais/15774-malhas.html. Terms of use available from https://biblioteca.ibge.gov.br/visualizacao/livros/liv102169.pdf.

This study was carried out with patients born in eco-geographically distinct mesoregions (Sertão, Agreste, Zona da Mata, and Metropolitan Region) of the state of Pernambuco, localized in the Northeast region of Brazil **(Fig 2)**. In the studied cohort, all included patients attending the referral PROCAPE hospital presented two positive serological tests for *T. cruzi* infection, had not received previous etiological treatment for CD, and met additional inclusion criteria. As shown in **Table 1**, 67% of patients being female and 33% male. The average age of the study group was 60.5 years, and most of these patients were over 45 years old. We observed significant differences when comparing three variables: age strata, monthly income and region of origin. For age strata ≤ 45 years compared to > 45 years, there were significant differences between the different clinical classifications (Chisq value = 0.006). In terms of monthly income most patients had a minimum monthly wage income (~US$300) and there were significant differences when comparing between clinical groups (Fisher = 0.025). The patients were from different geographic areas (Sertão, Agreste, Zona da Mata, and Metropolitan Region) of the State of Pernambuco. We observed that most patients were born in Zona da Mata (48.3%), followed by Sertão (18.8%), Agreste (16%), and Metropolitan Region (5.8%). Also, when comparing region of origin of the patients there were significant differences between clinical groups (Fisher < 0.001). Regardless of the patient’s region of origin, 88% of them lived in mud houses, primarily during childhood, and ~90% reported knowing the CD vector, and having a basic level of education (maximum 4 years), as shown in **Table 1**. As expected, since it was a criterion used for clinical classification, the left ventricular ejection fraction (LVEF%) also showed a statistically significant difference between the clinical groups of patients (Kruskal-Wallis < 0.001).

**Table 1:** Demographic, epidemiological, and clinical data of the group of non-etiologically treated Chagas disease patients in Pernambuco State, Brazil.

Variable	Non-CARD N = 57 (16.5)	CARD N = 232 (67%)			CARD/DIG N = 42 (12.1)	DIG N = 15 (4.3)	*p*-value
		B1 N = 125	B2 N = 14	C N = 93			
**Gender N = 346** Female N = 231 (67) Male N = 115 (33)	31 (54.4) 26 (45.6)	93 (74.4) 32 (25.6)	8 (57.1) 6 (42.9)	57 (61.3) 36 (38.7)	31 (73.8) 11 (26.2)	11 (73.3) 4 (26.7)	Chisq = 0.073
**Age (years) N = 346** ≤ 45 N = 52 (15) > 45 N = 294 (85)	18 (31.6) 39 (68.4)	17 (13.6) 108 (86.4)	1 (7.1) 13 (92.9)	9 (9.7) 84 (90.3)	4 (9.5) 38 (90.5)	3 (20) 12 (80)	Chisq = 0.006
**Ethnicity N = 321** White N = 50 (15.6) Black N = 35 (10.9) Mestizo N = 236 (73.5)	N = 52 14 (26.9) 5 (9.6) 33 (63.5)	N = 113 14 (12.4) 12 (10.6) 87 (77)	N = 14 1 (7.1) 0 13 (92.9)	N = 88 18 (20.4) 10 (11.4) 60 (68.2)	N = 40 2 (5) 7 (17.5) 31 (77.5)	N = 14 1 (7.1) 1 (7.1) 12 (85.8)	Fisher = 0.102
**Monthly income N = 288** Up to 1 MW N = 224 (77.8) 2-4 N = 43 (14.6) More than 5 N = 21 (7.3)	N = 48 41 (85.4) 7 (14.6) 0	N = 109 76 (69.7) 17 (15.6) 16 (14.7)	N = 12 9 (75) 3 (25) 0	N = 80 65 (81.3) 10 (12.5) 5 (6.2)	N = 28 26 (92.9) 2 (7.1) 0	N = 11 7 (63.6) 4 (36.4) 0	Fisher = 0.025
**Education N = 325** Up to 4 years N = 284 (87.4) More than 4 years N = 41 (12.6)	N = 54 47 (87) 7 (13)	N = 118 98 (83.1) 20 (16.9)	N = 12 11 (91.7) 1 (8.3)	N = 89 83 (93.3) 6 (6.7)	N = 39 33 (84.6) 6 (15.4)	N = 13 12 (92.3) 1 (7.7)	Fisher = 0.325
**Lived in mud houses N = 346** Positive N = 303 (87.6) Negative N = 41 (11.8)	N = 57 48 (84.2) 9 (15.8)	N = 125 109 (87.2) 16 (12.8)	N = 14 13 (92.9) 1 (7.1)	N = 91 84 (90.3) 7 (7.5)	N = 42 38 (90.5) 4 (26.7)	N = 15 11 (73.3) 4 (26.7)	Fisher = 0.318
**Know the vector N = 346** Positive N = 310 (89.6) Negative N = 36 (10.4)	N = 57 49 (86) 8 (14)	N = 125 110 (88) 15 (12)	N = 14 12 (85.7) 2 (14.3)	N = 93 85 (91.4) 8 (8.6)	N = 42 40 (95.2) 2 (4.8)	N = 15 14 (93.3) 1 (6.7)	Fisher = 0.637
**Region N = 346** Sertão N = 65 (18.8) Agreste N = 94 (27.2) Mata N = 167 (48.3) Metropolitan N = 20 (5.8)	N = 57 19 (33.3) 20 (35.1) 15 (26.3) 3 (5.3)	N = 125 25 (20) 19 (15.2) 74 (59.2) 7 (5.6)	N = 14 2 (14.3) 5 (35.7) 5 (35.7) 2 (14.3)	N = 93 10 (10.7) 33 (35.5) 46 (49.5) 4 (4.3)	N = 42 5 (11.9) 13 (31) 21 (50) 3 (7.1)	N = 15 4 (26.7) 4 (26.7) 6 (40) 1 (6.6)	Fisher < 0.001
**Clinical information N = 311** LVEF (%)	N = 53 66.8 ± 5.8	N = 109 65.9 ± 6.6	N = 13 52.1 ± 7.5	N = 93 40.4 ± 11.5	N = 38 57.7 ± 11.7	N = 8 68.4 ± 6.9	Kruskal-Wallis < 0.001

Abbreviations: non-CARD = Non-Cardiopathic; CARD = Cardiac form; DIG = Digestive form; CARD/DIG = Cardiodigestive form; N = Number of patients; LVEF = Left Ventricular Ejection Fraction. (%). The numbers in parentheses represent percentage.

Regarding clinical classification, out of the 346 patients included in this study 57 (16.5%) were classified as non-CARD (A), 125 as mild heart disease (B1), 14 as moderate heart disease (B2), and 93 as severe heart disease (C), therefore 232 (67%) were classified as CARD patients. In addition, 42 (12.1%) had a CARD/DIG form, and 15 (4.3%) patients were classified as having a DIG form (**Table 1**).

### Molecular diagnosis by conventional PCR

**Fig 3:**
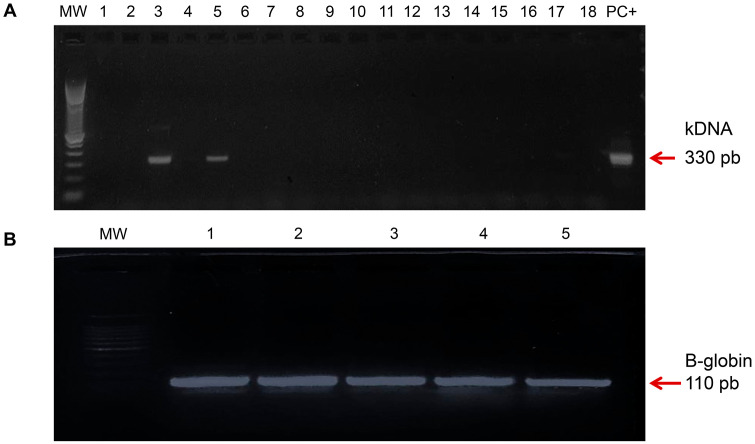
Representative 2% agarose gels for conventional PCR for Chagas disease diagnosis and β-globin gene. A. Representative amplification of *Trypanosoma cruzi* kDNA. Arrow indicates a band at approximately 330 bp (*T. cruzi* kDNA). MW, Molecular weight; Lane 1, NTC, no template control; Lanes 2-18 correspond to clinical samples of CD patients; PC +, positive control (TcII strain). B. Representative amplification of β-globin gene fragment of 110 bp in samples of serology-positive CD patients. Arrow indicates a band at approximately 110 bp (β-globin gene). MW, Molecular weight; Lanes 1 to 5 correspond to kDNA-negative clinical samples of CD patients.

Using DNA isolated from blood samples, we performed molecular diagnosis of the 346 samples included in the present study. Of these patients, 128 (37%) were positive for the presence of *T. cruzi* kDNA, revealed by the presence of the 330 bp fragment (**Fig 3A**). In parasite kDNA-negative samples, the detection of the human beta-globin gene revealed the satisfactory quality of the isolated DNA, as shown in the demonstrative figure revealed by the presence of the 110 bp fragment (**Fig 3B**).

**Fig 4:**
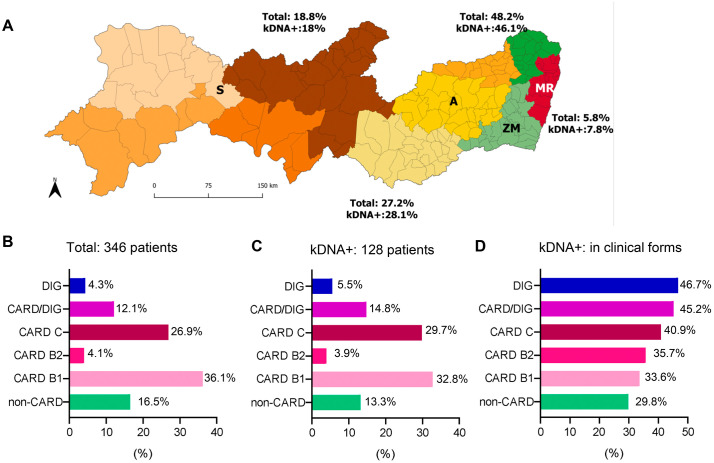
Distribution of patients with Chagas disease diagnosed by conventional PCR for *Trypanosoma cruzi* kDNA. **A.** Percentages of total patients diagnosed for CD (Total) and positive for *T. cruzi* kDNA (kDNA+) in the mesoregions of Pernambuco/Brazil. Map of Pernambuco shows: Brown, Sertão **(S)**; Yellow, Agreste **(A)**; Green, Zona da Mata (ZM); Red, Metropolitan Region (MR). Different shades of a color indicate geographically considered sub-areas within a mesoregion. **B.** Percentage of CD patients diagnosed by PCR for *T. cruzi* kDNA according to the different clinical forms of CD. **C.** Percentage of patients positive for *T. cruzi* kDNA in the different clinical forms of CD. **D.** Percentage of PCR positivity for *T. cruzi* kDNA in each clinical group of CD patients. The map was obtained from https://www.ibge.gov.br/geociencias/organizacao-do-territorio/malhas-territoriais/15774-malhas.html.

In the studied group of 346 patients, 65 (18.8%) were born in Sertão, 94 (27.2%) in Agreste, 167 (48.3%) in Zona da Mata, and 20 (5.8%) in the Metropolitan Region. The *T. cruzi* kDNA-positive samples were: 23 (18%) samples of patients born in Sertão, 36 (28.1%) in Agreste, 59 (46.1%) in Zona da Mata, and 8 (7.8%) in the Metropolitan Region (**Fig 4A**). Thus, we found a similar percentage of positivity for *T. cruzi* kDNA according to the original frequency of contribution of samples of each mesoregion to the studied group (**Fig 4A**), indicating that the conventional PCR positivity was not preferentially found in any region (Chisq value, *p* > 0.05). Further, the original group of 346 patients was composed of non-CARD patients 57 (16.5%), CARD patients (232, 67%), CARD/DIG patients (42, 12.1%), and DIG patients (15, 4.3%). According to severity, CARD group was composed of patients classified as B1 (125, 36.1%), B2 (14, 4.1%), and C (93, 26.9%), as shown in **Fig 4B** and **Table 2**. Next, the 128 patients (37%) positive for the presence of *T. cruzi* kDNA were clinically characterized as non-CARD patients (17, 13.3%); CARD patients (85, 66.4%), including B1 (42, 32.8%), B2 (5, 3.9%) C (38, 29.7%); CARD/DIG group (19, 14.8%); and DIG group (7, 5.5%), as shown in **Fig 4C** and **Table 2**. Again, the frequency of PCR^+^ for *T. cruzi* kDNA reflected the input of each clinical group in the original studied population. Further, considering the clinical parameters, PCR positivity for *T. cruzi* kDNA in each group was as follow: non-CARD patients (17/57, 29.8%); CARD group (85/232, 36.6%), composed of B1 (42/125, 33.6%), B2 (5/14, 35.7%) C (38/93, 40.9%) patients; CARD/DIG group (19/42, 45.2%); and DIG group (7/15, 46.7%), as shown in **Fig 4D**. Such differences between clinical groups were significantly different (Chisq value, *p* < 0.001). Although these data suggested an increasing trend in PCR positivity for *T. cruzi* kDNA as the CARD form is aggravated, the trend test was not significant in the group presenting the mixed form (CARD/DIG) (Cochran-Armitage, *p* = 0.20).

**Table 2:** Samples positive for *T*. *cruzi* kDNA and genotyped for DTU in clinically classified Chagas disease patients in the State of Pernambuco, Brazil.

Clinical form	Total N = 346	kDNA PCR^+^ N = 128	Genotyped N = 49	Single DTU N = 43	Mixed DTU N = 6	Inconclusive N = 36
**Non-CARD**	57 (16.5%)	17 (13.3%)	3 (6.1%)	3	0	6
**CARD**	232 (67.1%)	85 (66.4%)	36 (73.5%)	30	6	23
**CARD/DIG**	42 (12.1%)	19 (14.8%)	8 (16.3%)	8	0	4
**DIG**	15 (4.3%)	7 (5.5%)	2 (4.1%)	2	0	3

Abbreviations: non-CARD = Non-Cardiopathic; CARD = Cardiac form; DIG = Digestive form; CARD/DIG = Cardiodigestive form; N = Number of patients; DTU = Discrete Typing Units.

### Molecular DTU genotyping of *T. cruzi* in blood samples of CD patients

Molecular DTU genotyping was performed in blood samples positive for *T. cruzi* kDNA. Patient samples were subjected to a multilocus intraspecific *T*. *cruzi* DTU genotyping approach, enabling the molecular characterization directly from blood samples. Out of the 128 samples positive for *T. cruzi* kDNA, 85 (66%) amplified target products, according to the adopted decision algorithm (**Fig 1**), allowing genotyping of 49 samples (38%), including mixed infections in 6 samples. Although the genotyping procedures were repeated at least twice, and reference strains as DTU controls were included and allowed confidence, 36 patient samples remained inconclusive for DTU genotyping (**Table 2**). For patients who could be effectively genotyped, we evaluated potential differences between the clinical classification of patients and each of the genotyped DTUs (single DTU or mixed DTU), which showed no significant differences (Fisher´s = 0.25 for single DTUs and Fisher´s = 0.69 for mixed DTUs).

**Fig 5:**
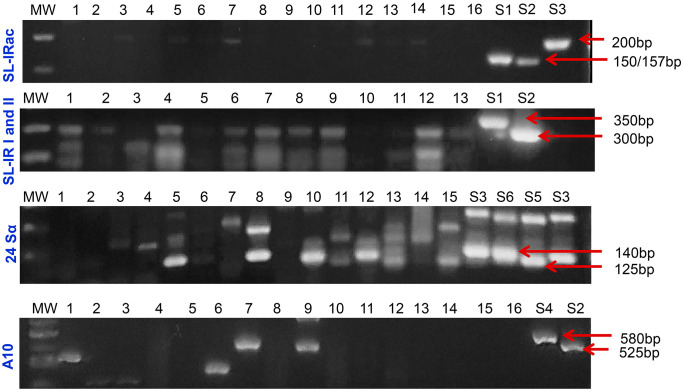
Agarose gels for different targets of *Trypanosoma cruzi* in blood samples of Chagas disease patients. Agarose gels with products obtained after conventional PCR for the different targets of *T. cruzi* for the classification of DTUs in blood samples of CD patients. Abbreviations: MW, molecular weight marker (100 bp); Lanes 1-16, clinical samples of CD patients; S, Strain; S1-Dm28c (TcI); S2-CL Brener (TcVI); S3-INPA 4167 (TcIV); S4-Y (TcII); S5-INPA 3663 (TcIII); S6-LL014 (TcV); bp, base pairs.

**Fig 5** shows representative images of agarose gels with positive amplifications for SL-IRac, SL-IR I and II, 24Sα rDNA, and A10 targets. The algorithm data supporting the DTU classification of the 49 samples is shown in **S3 Table**. Following the adopted algorithm, these data show that the most common DTU was TcV (16), followed by TcIII (13), TcIV (7), TcVI (3), TcI (3), and TcII (1). We also identified mixed infections in six patient samples, three of which contained TcIII + TcIV, two samples contained TcI + TcV, and one contained TcIII + TcV. Furthermore, 13 patient samples showed positive amplification for SL-IR I and II, but negative for 24Sα rDNA, and were reported as TcII/V/VI. Interestingly, 7 patient samples were classified as bearing the TcII/TcVI *T. cruzi* genotypes, and 11 samples as carrying the TcIII/TcIV *T. cruzi* genotypes (**Table 3**). Furthermore, five patient samples, in addition to identifying one or two (mixed) *T. cruzi* genotypes, also revealed the presence of other DTU genotypes, rendering the results inconclusive (**Table 3**).

**Table 3:** Samples of DC patients genotyped for DTU distributed by attributes local of birth in geographical mesoregions of the State of Pernambuco, Brazil.

Genotyped DTU	Total	Sertão 13 (15.3)	Agreste 25 (29.4)	Zona da Mata 39 (45.9)	Região Metropolitana 8 (9.4)
TcI	3 (7)	0	1 (7.1)	1 (5.9)	1 (16.7)
TcII	1 (2.3)	0	1 (7.1)	0	0
TcIII	13 (30.2)	3 (50)	4 (28.6)	6 (35.3)	0
TcIV	7 (16.3)	1 (16.7)	2 (14.3)	2 (11.8)	2 (33.3)
TcV	16 (37.2)	2 (33.3)	5 (35.7)	7 (41.2)	2 (33.3)
TcVI	3 (7)	0	1 (7.1)	1 (5.9)	1 (16.7)
**Mixed infections**					
TcI + TcV	2 (33.3)	0	0	2 (50)	0
TcIII + TcIV	3 (50)	1 (100)	1 (100)	1 (25)	0
TcIII + TcV	1 (16,7)	0	0	1 (25)	0
**Partially genotyped**					
TcI + TcII/TcV/TcVI	2 (5.6)	0	0	2 (11.1)	0
TcI + TcII/TcV/TcVI + TcIII/TcIV	1 (2.8)	1 (16.7)	0	0	0
TcI + TcII/TcVI	1 (2.8)	1 (16.7)	0	0	0
TcII/TcV/TcVI	13 (36.1)	1 (16.7)	3 (30)	8 (44.4)	1 (50)
TcII/TcVI	7 (19.4)	0	4 (40)	3 (16.7)	0
TcIII/TcIV	11 (30.6)	3 (50)	3 (30)	4 (22.2)	1 (50)
TcV + TcII/TcVI	1 (2.8)	0	0	1 (5.6)	0

Information for each variable is represented by total count N then followed by the percentage expressed between parentheses (%). Percentages refer to each group of DTUs genotyped: Single DTUs, mixed DTUs infections and partially genotyped DTUs.

### Geographic distribution of patient samples genotyped for *T. cruzi* DTU

**Fig 6:**
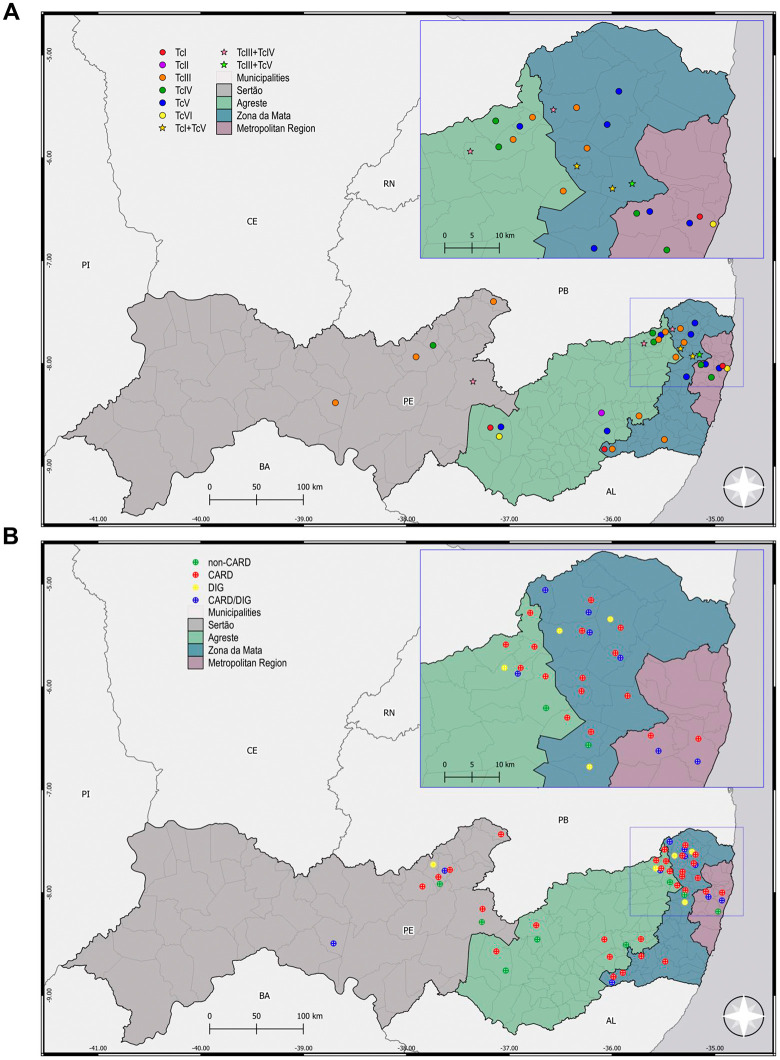
Maps showing the spatial distribution of clinically classified CD patients and DTUs *of Trypanosoma cruzi* in the different mesoregions of Pernambuco/Brazil. A. Map, geographic distribution of samples from patients classified into *T. cruzi* DTUs TcI-TcVI. B. Map, geographic distribution of patients classified according to clinical forms of CD and submitted to genotypic characterization of *T. cruzi*. This map was created using QGIS 3.34 ‘Prizren’ version software and cartographic bases obtained from the Brazilian Institute of Geography and Statistics https://www.ibge.gov.br/geociencias/organizacao-do-territorio/malhas-territoriais/15774-malhas.html).

As registered in the questionnaire answered by the CD patients included in this study, most of them were born and grew up in rural environments, living in mud houses (88%) with the presence of the triatomine vector (79%), as described in **Table 1**. Considering their life report indicating birthplace, rare report of migration between mesoregions (14.1%), epidemiological traits in the State of Pernambuco [31,32], and supported by previous works [12,43], we assumed that these patients were infected in early childhood, therefore considering the birthplace as local of infection, as shown in **Fig 6**.

In addition, when the samples identified by DTU were distributed according to the patients’ place of birth in the mesoregions (**Figs 6A** and **S1**), Agreste was the only mesoregion in which we identified samples of all described DTUs. The most frequent was TcV (5), followed by TcIII (4), TcIV (2), and TcI, TcII, and TcVI (1 of each), as well as a mixed TcIII + TcIV infection. Zona da Mata was the region where we detected the most significant number of successfully genotyped *T. cruzi* DTU samples (21). Once again, the most prevalent DTUs were TcV (7) and TcIII (6), followed by TcIV (2), TcI (1), and TcVI (1), as well as mixed infections of TcI + TcV (2), TcIII + TcIV (1) and TcIII + TcV (1). In the Metropolitan Region, we found samples carrying the TcIV (2), TcV (2), TcI (1), and TcVI (1) DTU genotypes. In the Sertão mesoregion, we identified samples characterized as TcIII (3), TcIV (1), and TcV (1), in addition to a sample showing mixed infection with TcIII + TcIV (**Fig 6A** and **Table 3**). Further, five samples were partially classified (TcII/TcVI – 2; TcIII/TcIV – 3), and potentially mixed infection with TcI + TcII/TcV/TcVI + TcIII/TcIV DTU profile (**S1 Fig** and **Table 3**). When evaluating the association between genotyped DTUs (single DTUs, mixed infections and partially genotyped DTUs) and the different mesoregions of DC patients’ birth there were no significant differences for single DTUs genotyped (Fisher = 0.87) or partially genotyped DTUs (Fisher = 0.4).

### Relationship between *T. cruzi* DTU genotypes and clinical manifestations

**Fig 6B** shows the distribution of clinically classified patients as non-CARD, CARD, CARD/DIG, and DIG born in different mesoregions of the state of Pernambuco (Sertão, Agreste, Zona da Mata, and Metropolitan Region). Next, we investigated the association of *T. cruzi* DTU genotypes found in blood samples with the clinical form of CD patients. **Table 2** summarizes the quantitative data obtained after sample genotyping, showing that only in the CARD group were mixed infections identified (6). Additionally, the largest number of genotyped samples, 36/49 (73.5%), was in the CARD form. **Table 4** presents data of samples genotyped for DTU in clinically classified Chagas disease patients in the State of Pernambuco. In patients classified as non-CARD, we found TcIV (1), TcV (1), and TcVI (1), while five samples were partially classified (TcII/TcVI – 2; TcIII/TcIV – 3), and potentially mixed infection with TcI + TcII/TcV/TcVI + TcIII/TcIV DTU profile was detected. When we analyzed the *T. cruzi* genotypes found in samples of CARD patients, we observed a higher number of samples bearing the TcV (13) and TcIII (10), while TcIV (3), TcI (2), TcII (1), and TcVI (1) were also found. Interestingly, mixed infections with TcIII + TcIV (3), TcI + TcV (2), and TcIII + TcV (1) were also detected in samples of CARD patients. In CARD/DIG form, we observed samples carrying the TcIII (3), TcIV (3), TcI (1), and TcVI (1) genotypes. Further, in samples of patients with the CARD/DIG form, four samples were partially classified as TcII/TcV/TcVI (3) and TcII/TcVI (1). Although the number of DIG form patients was lower, we identified the TcV DTU (2), and three other samples were partially classified as TcII/TcV/TcVI (2) and TcIII/TcIV (1). Though we have identified a higher number of *T. cruzi* DTUs and mixed infections in CARD patients, no association has been found between a specific DTU and clinical form and/or course in this study. When evaluating the association between DTU genotypes (single DTUs, mixed infections and partially genotyped DTUs) and clinical classification of DC patients, there were no significant differences for single DTUs genotyped (Fisher = 0.09) or partially genotyped DTUs (Fisher = 0.50).

**Table 4:** Samples genotyped for DTU in clinically classified Chagas disease patients in the State of Pernambuco, Brazil.

Genotyped DTU	Total	Non-CARD	CARD	CARD/DIG	DIG
TcI	3 (7)	0	2 (6.7)	1 (12.5)	0
TcII	1 (2.3)	0	1 (3.3)	0	0
TcIII	13 (30.2)	0	10 (33.3)	3 (37.5)	0
TcIV	7 (16.3)	1 (33.3)	3 (10)	3 (37.5)	0
TcV	16 (37.2)	1 (33.3)	13 (43.3)	0	2 (100)
TcVI	3 (7)	1 (33.3)	1 (3.3)	1 (12.5)	0
**Mixed infections**					
TcI + TcV	2 (33.3)	0	2 (100)	0	0
TcIII + TcIV	3 (50)	0	3 (100)	0	0
TcIII + TcV	1 (16,7)	0	1 (100)	0	0
**Partially genotyped**					
TcI + TcII/TcV/TcVI	2 (5.6)	0	2 (8.7)	0	0
TcI + TcII/TcV/TcVI + TcIII/TcIV	1 (2.8)	1 (16.7)	0	0	0
TcI + TcII/TcVI	1 (2.8)	0	1 (4.3)	0	0
TcII/TcV/TcVI	13 (36.1)	0	8 (34.8)	3 (75)	2 (66.7)
TcII/TcVI	7 (19.4)	2 (33.3)	4 (17.4)	1 (25)	0
TcIII/TcIV	11 (30.6)	3 (50)	7 (30.4)	0	1 (33.3)
TcV + TcII/TcVI	1 (2.8)	0	1 (4.3)	0	0

Information for each variable is represented by total count N then followed by the percentage expressed between parentheses (%). Percentages refer to each group of DTUs genotyped: Single DTUs, mixed DTUs infections and partially genotyped DTUs. Abbreviations: non-CARD = Non-Cardiopathic; CARD = Cardiac form; DIG = Digestive form; CARD/DIG = Cardiodigestive form.

**Table 5** shows the distribution in the mesoregions of Pernambuco of samples of patient classified in clinical forms of Chagas disease according to genotyped DTU. Considering the genotyped samples, among the 11 patients born in Sertão, 2 were clinically classified as non-CARD [TcIII/TcIV, TcI + TcII/TcV/TcVI + TcIII/TcIV], 6 as CARD [TcIII (2), TcIV, TcV, TcIII + TcIV, TcIII/TcIV, TcI + TcII/TcVI], 2 as CARD/DIG [TcIII, TcII/TcV/TcVI] and 1 as DIG [TcIII/TcIV]. In this study, of the 22 patients born in Agreste, 5 were clinically classified as non-CARD [TcV, TcVI, TcII/TcVI (2), TcIII/TcIV], 15 as CARD [TcI, TcII, TcIII, TcIV, TcV (4), TcIII + TcIV, TcII/TcVI (2), TcIII/TcIV (2), TcII/TcV/TcVI], 1 as CARD/DIG [TcIV] and 1 as DIG [TcII/TcV/TcVI]. In addition, of the 37 patients who were born in Zona da Mata, 1 was clinically classified as non-CARD [TcIII/TcIV], 26 were characterized as CARD [TcI, TcIII (4), TcIV, TcV (5), TcI + TcV, TcIII + TcIV, TcIII + TcV, TcII/TcVI (2), TcIII/TcIV (3), TcII/TcV/TcVI (5), TcI + TcII/TcV/TcVI TcV + TcII/TcVI], 7 as CARD/DIG [TcIII (2), TcIV, TcVI, TcII/TcVI, TcII/TcV/TcVI (2)] and 3 as DIG [TcV (2), TcII/TcV/TcVI}. Lastly, of the 7 patients born in Região Metropolitana, 1 was classified as non-CARD [TcIV], 4 as CARD [TcV, TcVI, TcIII/TcIV, TcII/TcV/TcVI], and 2 as CARD/DIG [TcI, TcIV]. Altogether, these data reinforce the non-association of clinical form of CD with the genotype DTUs in the mesoregions of the State of Pernambuco.

**Table 5:** Distribution of samples of patient classified in clinical forms of Chagas disease according to genotyped DTU in the mesoregions of Pernambuco (PE), Brazil.

Local of birth in PE (Mesoregion)	Number of patients	Clinical classification	*T. cruzi* genotype (DTU)
**Sertão** 11	2 6 2 1	non-CARD CARD CARD/DIG DIG	TcIII/TcIV, TcI + TcII/TcV/TcVI + TcIII/TcIV **TcIII (2), TcIV, TcV, TcIII + TcIV,** TcIII/TcIV, TcI + TcII/TcVI **TcIII**, TcII/TcV/TcVI TcIII/TcIV
**Agreste** 22	5 15 1 1	non-CARD CARD CARD/DIG DIG	**TcV, TcVI,** TcII/TcVI (2), TcIII/TcIV **TcI, TcII, TcIII, TcIV, TcV (4),** TcIII + TcIV, TcII/TcVI (2), TcIII/TcIV (2), TcII/TcV/TcVI **TcIV** TcII/TcV/TcVI
**Zona da Mata** 37	1 26 7 3	non-CARD CARD CARD/DIG DIG	TcIII/TcIV **TcI, TcIII (4), TcIV, TcV (5),** TcI + TcV, TcIII + TcIV, TcIII + TcV, TcII/TcVI (2), TcIII/TcIV (3), TcII/TcV/TcVI (5), TcI + TcII/TcV/TcVI TcV + TcII/TcVI **TcIII (2), TcIV, TcVI, TcII/TcVI,** TcII/TcV/TcVI (2) TcV (2), TcII/TcV/TcVI
**Região Metropolitana** 7	1 4 2	non-CARD CARD CARD/DIG	**TcIV** **TcV, TcVI,** TcIII/TcIV, TcII/TcV/TcVI **TcI, TcIV**

Patients born in mesoregions (N = 346): Sertão (N = 65, 18.8%), Agreste (N = 94, 27.2%), Mata (N = 167, 48.2%), Metropolitan (N = 20, 5.8%); Abbreviations: non-CARD = Non-Cardiopathic; CARD = Cardiac form; DIG = Digestive form; CARD/DIG = Cardiodigestive form. In DTUs: bold lettering refers to single DTUs detected, italics lettering to mixed DTUs infection.

### Gender and identification of DTU

Lastly, due to concerns about *T. cruzi* infection in women of childbearing age [44,45] and knowing that TcV DTU appears to be more related to the congenital form of transmission [46] we questioned a possible association between different parasite DTUs and gender. Among the samples that amplified for the target products according to the adopted algorithm (49 patients), 32 (65.3%) were female and 17 (34.7%) were male (**S3 Table**), reflecting the differential contribution of genders to this study (67% female; 33% male). As shown in **S3 Table**, in samples of female patients we detected as unique infections a predominance of TcV (11) and TcIII (10), followed by TcI (2), TcV (4), TcVI (1), and mixed infections with TcI + TcV (1), TcIII + TcIV (2), and TcIII + TcV (1). In the group of male patients, we also identified unique infections a predominance of TcV (5), and TcIII (3), as well as mixed infections of TcI + TcV (1), and TcIII + TcIV (1). Subsequently, we considered the DTU identified in mixed infections as unique and observed, in the group of female patients, the same predominance of TcV (13, 40.6%), TcIII (13, 40.6%), and TcIV (6, 18.8%). In the group of male patients, TcV also prevailed (6, 42.8%), followed by TcIV (4, 28.6%), and TcIII (4, 28.6%). Thus, in this study population, TcV DTU was prevalent in samples from both female and male CD patients (**S3 Table**).

## Discussion

Here, studying a cohort born and resident in the state of Pernambuco/Brazil attending a referral hospital, we employed a multilocus intraspecific *T. cruzi* genotyping approach, attempting to establish an association with its distribution in the distinct ecogeographic areas and clinical CD forms. Further, patients did not receive etiological treatments to avoid a putative selection of DTUs, which may be differentially susceptible to trypanocidal drugs [27]. These data show that, although not frequently found in human infections in Brazil, DTUs TcIII and TcV, and TcIV were predominantly detected in patients born in Pernambuco. Further, all analyzed DTUs (TcI-TcVI) were only detected in the blood of CD patients born in Agreste. In the characterized samples, DTUs TcV and TcIII prevailed. Therefore, we provided new information regarding the distribution of *T. cruzi* DTU genotypes present in a unique region of Brazil. Lastly, these data could not disclose an identifiable association between *T. cruzi* DTU detected in circulating blood and clinical forms of CD, thereby strengthening the challenge of comprehending the parasite-host interplay in this complex disease.

In the studied cohort, a large proportion of patients were born and lived their childhood in the same mesoregions of the state of Pernambuco. Regardless of origin, most of them lived in mud houses and informed the presence of *T*. *cruzi* vectors in their homes during childhood, common traits in areas endemic for CD [47], reinforcing the assumption of exposure to infection in this early period of life. Therefore, we assumed the birthplace as the site of infection, as previous works [12,43]. Importantly, regardless of the birthplace and clinical form of CD, most of the patients had similar socioeconomic traits such as a low monthly income and basic education level, as described for most of the vulnerable populations exposed to *T. cruzi* infection [48–51]. Further, most of the patients were over 45 years old. The average age of the studied group was over 60 years old, reinforcing the impact of vector control measures implemented in Brazil, which led to an overall reduction in the incidence of CD, particularly among younger individuals [1,52]. Also, most of the patients were female, as reported in other studies [47,51,53].

Here, most patients were native to Zona da Mata, followed by Sertão, Agreste, and Metropolitan Region, i. e., all mesoregions were represented. Previously, higher rates of chronic CD were reported in Zona da Mata and Sertão, both regions characterized by a predominance of rural areas, a low human development index, and high mortality rates [47]. In Argentina, a study revealed the predominance of vector transmission in rural areas transitioning to a semi-arid climate [54], characteristics like those of the regions in this study. Therefore, identifying areas with higher incidence values can help in the implementation of epidemiological and entomological surveillance actions, as well as support the integration of assistance services and decentralization of specialized health services [47].

In this study, out of the 346 patient samples submitted to molecular diagnosis by PCR, 37% were positive for *T. cruzi* kDNA. PCR for detecting parasite DNA may be more suitable in cases of recent infections, CD reactivation, and treatment failure [55–57]. In the chronic phase of CD, PCR performance may be variable, due to the intermittent parasitemia [58,59]. However, our work license allowed only one blood withdrawal per patient. Similarly, a study involving 150 seropositive CD patients found a PCR positivity of 31.3% [60]. A study that recruited patients from diverse Brazilian states attending a referral hospital in Rio de Janeiro showed that 53.8% of patient samples were positive for *T. cruzi* sat-DNA by qPCR [12]. Indeed, the sensitivity of PCR diagnosis in chronic CD patients can vary according to the sample type, isolation method, molecular target and parasite genotype [59,61]. Moreover, the percentage of conventional kDNA PCR positivity within the mesoregions of Pernambuco only reflected the initial contribution of the frequency of patient input to each mesoregion. Regarding the clinical form and PCR positivity for *T. cruzi* kDNA, these data indicate a trend of increased detection comparing the non-CARD with the CARD and the severity of the cardiac form, as well as in the CARD/DIG and DIG forms; however, these differences were not statistically significant. A previous study with Argentinean patients showed higher PCR positivity for the nuclear DNA fragment E13 in CD patients with heart disease (86%) when compared to patients without heart injury (21%) [62]. Also, a study involving 499 Brazilian CD patients found that CARD patients had a higher PCR positivity for kDNA (75.2%) compared to non-CARD patients (51.3%) [63]. Notably, a study with 333 chronic CD patients reported that 41.1% of them were positive for *T. cruzi* sat-DNA PCR and highlighted that trypanocidal treatment was a strong protective factor against positive PCR for *T. cruzi* [64], reinforcing data of the Benefit Study [65]. It is worth mentioning that the patients included in this study did not receive treatment with trypanocidal drug prior to blood sampling. Particularly, our concern was the differential susceptibility of parasite DTUs to etiological treatment. A study conducted with chronically *T. cruzi*-infected Latin American immigrants living in Spain showed that 57% of patients presented different DTUs in circulating blood before and after treatment [66]. Altogether, these data reinforce our premise for studying patients who have not received previous etiological treatment for CD. Further, we did not use *ex-vivo* selection such as xenodiagnoses and hemoculture, methods that may allow selective growth of parasite strains [67]. However, one should consider that chronic infection could potentially allow decades of in-host selection due to host-parasite interactions in different cell types and tissues. Further, we sampled blood, and parasites of different DTUs may show different tissue tropism [68]. Therefore, one cannot rule out the inevitable impossibility of obtaining all the DTUs that these patients may have experienced throughout their lives, contributing to the clinical outcome.

For *T*. *cruzi* molecular genotyping, we used conventional PCR with pre-established targets and algorithms [29]. Of the 128 positive samples for parasite kDNA, 85 (66%) amplified for at least one target and 49 samples were characterized in DTUs (38%), including 6 samples that presented mixed infections. Even after repetition, the remaining patient samples (36) persisted inconclusively for DTU genotyping. Here, *T. cruzi* genotyping was performed directly from DNA extracted from blood samples, aiming to avoid strain selection during parasite isolation and to detect mixed infections in the samples, as previously proposed [12]. It is also important to highlight the previously described low parasite load in patients with chronic Chagas’ heart disease [69], challenging parasite DNA detection. Based on the algorithm used, these data revealed a higher frequency of TcV DTU, followed by TcIII, TcIV, TcVI, TcI, and TcII. Furthermore, we identified 6 samples with mixed infections composed of TcIII + TcIV, TcI + TcV, and TcIII + TcV. Thirteen samples were shown to contain TcII/TcV/TcVI, TcII/TcVI, and TcIII/TcIV genotypes, while 5 samples with mixed infections also presented other genotypes that were not conclusively characterized. Indeed, some DTUs are closely related, and TcII, TcV, and TcVI are sometimes collectively classified as “non-TcI” due to their genetic proximity [70]. In the future, the use of other genotyping tools may aid in the identification of these strains. Altogether, these data suggest that several CD patients were infected with different *T. cruzi* strains, emphasizing the possibility of reinfections. Indeed, in this study group, most patients reported knowing the vector and, in some cases, the persistent presence of triatomine vectors in their homes during childhood, supporting the notion that they were exposed to eco-epidemiological conditions that favored infection and reinfection(s).

The study population of this work was born in the different mesoregions of the State of Pernambuco (Sertão, Agreste, Zona da Mata, and Metropolitan Region). Agreste, an area of ecological transition from semi-arid to tropical Atlantic Forest, was the mesoregion in which samples were identified with all TcI-TcVI DTUs, with prevalence of TcV and TcIII, and with the presence of mixed infection. Also, in samples from patients born in the Zona da Mata region, the group with the most significant number of genotyped samples, TcV and TcIII were the most frequently identified DTUs. These DTUs were also detected in the Sertão region, while TcIII but not TcV was identified in the Metropolitan Region. These results also reveal the presence of other DTUs and mixed infections in these mesoregions; however, the detection of TcIII and TcV in patient samples is a notable finding. Despite the previously reported predominance of TcII and TcVI in the domestic cycle of *T*. *cruzi* transmission in Brazil [9,11,12], the present study identified TcV and TcIII as the most prevalent DTUs in chronic CD patients born in the State of Pernambuco, Northeastern Brazil. TcV is predominant in the Gran Chaco of the Southern Cone (Peru, Bolivia, northern Chile, and Argentina), as well as Paraguay and Uruguay. There are also some records of TcV in the extreme south of Brazil and Argentina [11,16]. TcIII is rarely associated with human infections in Brazil and has been described in only a few acute cases in the Amazon region [22]. Interestingly, TcIII was also identified in 3 patients with the indeterminate form of CD in the State of Rio Grande do Norte [23], also located in Northeast Brazil. Furthermore, a study identified TcIII and TcIV in samples collected from mammals across five Brazilian biomes, including Caatinga [21], a vegetation type found in the mesoregions of Agreste and Sertão of Pernambuco. In triatomines collected in the State of Pernambuco, mostly in the intradomicile of Agreste area, the presence of TcIII/TcIV and TcI has been detected in samples of *Panstrogylus megistus* and *P. lutzi*, though only the DTU TcI was identified in samples of *T. pseudomaculata* and *T. brasiliensis* [71]. More recently, TcV and TcI were found as the most prevalent DTUs in either single or mixed infections in *Triatoma braziliensis*, *T*. *pseudomaculata,* and *Rhodnius* sp. collected in the Center-North mesoregion of Piauí state, also located in Northeast Brazil [72]. These findings reveal the persistent risk of vector transmission in the domestic cycle and the potential for human infection by the same parasite DTUs detected in this group of patients, which were probably infected more than 50 years ago. Altogether, these data reinforce the need for permanent vector surveillance, house improvement, and information/educational approaches [73] as strategies to control CD in the State of Pernambuco and in neighboring states of the Brazilian Northeast.

Finally, we tried to associate the genotypes identified in the blood samples of patients with distinct clinical forms of chronic CD. In the present study, patients with the CARD form of CD were more frequent compared to patients classified as non-CARD, which may reflect the fact that this work was conducted at a referral center specializing in cardiology services, unlike studies conducted in other contexts [53]. Most of the samples with classified DTUs (73.5%) were CARD patients, which may reflect the original input of this group of patients in the present study. Interestingly, infections with more than one DTU were identified only in patients with heart disease, primarily TcV and TcIII, although TcIV, TcI, TcII, and TcVI were also detected. A reduced number of non-CARD, DIG, and CARD/DIG patient samples, which may also reflect the reduced number of samples of these groups to the cohort studied, were identified or partially classified. A reduced number of samples from non-CARD, DIG, and CARD/DIG patients, which may also reflect the reduced number of samples from these groups in the scientific cohort, were identified or partially detailed. A study conducted in Argentina, with a small number of patients with digestive (18 patients) and cardiodigestive (20 patients) forms, detected TcV DTU in samples from 4 CARD/DIG patients and 2 DIG patients, in addition to TcVI in 1 CARD/DIG patient and 1 DIG patient [16], like the DTUs identified in patients with these clinical forms in our study. TcII is the most frequently found DTU in genotyping studies conducted in Brazil, being associated with severe chronic cardiac and digestive conditions [9]. However, TcII has also been found in samples from patients with chronic cardiac conditions in countries such as Argentina, Bolivia, and Chile [13,15,43]. Here, only one sample was identified as infected with the TcII genotype in a patient with CARD form, but the possibility of infection by this genotype in samples from patients with inconclusive genotyping results cannot be ruled out. Furthermore, the characteristic that draws attention to TcV is its potential association with the congenital transmission of *T*. *cruzi* in Brazil, Bolivia, Chile, Paraguay, and Uruguay [15,74]. In this study, TcV was found in patients with or without cardiac and/or digestive damage. It is worth mentioning that in the studied cohort, composed mostly of women, this DTU was the most frequently identified, raising an alert to possible cases of congenital transmission in the studied regions. Furthermore, we do not rule out the possible difference in DTUs present in blood and tissue, based on a pioneering study that analyzed samples of cardiac and esophageal tissue from patients with chronic CD, observing distinct *T. cruzi* kDNA signatures in both organs, indicating a differential tissue distribution of distinct populations of *T. cruzi* [68]. In this study, although the elevated numbers of infections by single and mixed DTUs have been identified in patients with CARD form, there is no association between a specific DTU and the clinical form and/or severity of heart disease. It is also important to emphasize that the clinical manifestations of Chagas disease result from a complex interaction between the genetic characteristics of both the host and the parasite.

Here, we highlight a potential underreporting of DTUs that circulate within the domestic cycle of CD in Brazil. We believe that advances in molecular methodologies, which enable more precise classification, allow for a deeper understanding of the role of parasite diversity in chronic CD outcomes and its actual distribution in the Brazilian territory. Altogether, this study reinforces the need for studies that include more patients and the indication of sequential blood collection during routine medical follow-up for CD patients to increase the chance of identifying parasite DTUs. Here, we advocate for permanent surveillance of vectors and blood, house improvement, and the adoption of information/education strategies to control CD in environments where humans are in constant contact with potential sources of infection.

### Potential clinical implications

The present findings show the predominance of TcV DTU in patients with chronic CD in the state of Pernambuco. Associated with the presence of TcV in triatomine vector in the state of Piauí [72], also in Northeastern Brazil, we draw attention to the possible relationship between this DTU and the congenital form of transmission of CD [15,74], therefore advocating for testing and monitoring of women of childbearing age and/or pregnant women living or coming from endemic regions, to offer adequate treatments and prevent CD in children. Furthermore, we emphasize the importance of epidemiological surveillance and other genotypic characterization studies to accurately determine the geographic distribution of *T. cruzi* DTUs in Brazil.

### Limitations

One of the significant limitations of molecular biology studies with patients with chronic Chagas disease is the low parasite load at this stage of the disease. Furthermore, the collection of additional clinical samples, which would have allowed us to attempt to increase PCR positivity through serial collections, was not permitted, as these patients are already regularly monitored and frequently undergo routine examinations. We also considered the limitations of the methodology used for genotyping *T. cruzi*, which, despite presenting some inconclusive results, allowed us to characterize this parasite directly from blood samples, without the need for *ex-vivo* parasite growth, which could lead to the selection of strains. Furthermore, despite the predominance of TcV and TcIII DTUs found in patients’ samples, we cannot rule out infections with other DTUs in the samples that were partially classified. Furthermore, we emphasize the possibility that different types of parasites may reside in tissues. However, we would like to notify about a possible underreporting of DTUs circulating in the Brazilian territory, more specifically, in the state of Pernambuco, Northeast Brazil.

## Supporting information

S1 Table Primer sequences and cycling conditions used for the genotypic characterization of *Trypanosoma cruzi.* (DOCX)

S2 Table Concentrations and volumes used in PCR reactions for genotypic characterization of *Trypanosoma cruzi.* (DOCX)

S3 Table Data of non-etiologically treated patient samples positive for *Trypanosoma cruzi* kDNA and genotyped for DTU distributed according to birthplace (Pernambuco, Brazil) and clinical form. (DOCX)

S1 Fig Maps showing the spatial distribution of all samples classified in DTUs *of Trypanosoma cruzi* in the different mesoregions of Pernambuco/Brazil. This map was created using QGIS 3.34 ‘Prizren’ version software and cartographic bases obtained from the Brazilian Institute of Geography and Statistics https://www.ibge.gov.br/geociencias/organizacao-do-territorio/malhas-territoriais/15774-malhas.html.
(TIF)
