# Supplementary material for: Trypanosoma cruzi DTU parasite diversity and clinical outcomes in mesoregions of the Northeast Brazilian State of Pernambuco
Source: PLoS Negl Trop Dis. 2026 Feb 13;20(2):e0013996. doi: 10.1371/journal.pntd.0013996 (PMC12923128; doi:10.1371/journal.pntd.0013996)
Supplement: S1 Table — (DOCX) [file pntd.0013996.s001.docx]

**S1 Table. Primer sequences and cycling conditions used for the genotypic characterization of *Trypanosoma cruzi*.**

| **Target** | **Primers** | **Sequences** | **Thermocycling steps** |
| --- | --- | --- | --- |
| **kDNA** | 121  122 | AAATAATGTACGGG(T/G)GAGATGCATGA  GGTTCGATTGGGGTTGGTGTAATATA | 94°C – 3 minutes;  98°C – 1 min, 64°C – 2 min (2 times);  94°C – 1 min, 64°C – 1 min (38 times);  72°C – 10 min. |
| **β-globin** | PCO3  PCO4 | ACACAAACTGTGTTCACTAGC  CAACTTCATCCACGTTCACC | 94°C – 3 minutes;  98°C – 1 min, 64°C – 2 min (2 times);  94°C – 1 min, 64°C – 1 min (38 times);  72°C – 10 min. |
| **SL-IRac** | UTCC  TCac | CGTACCAATATAGTACAGAAACTG CTCCCCAGTGTGGCCTGGG | 94°C – 3 minutes;  94°C – 30 sec, 70°C – 30 sec and 72°C – 30 sec (3 times);  94°C – 30 sec, 68°C – 30 sec and 72°C – 30 sec (3 times);  94°C – 30 sec, 66°C – 30 sec and 72°C – 30 sec (4 times);  94°C – 30 sec, 64°C – 30 sec and 72°C – 30 sec (4 times);  94°C – 30 sec, 62°C – 30 sec, and 72°C – 30 sec (36 times);  72 – 10 min. |
| **SL-IR I and II** | TCC  TC1  TCC | CCCCCCTCCCAGGCCACACTG GTGTCCGCCACCTCCTTCGGGCC CCTGCAGGCACACGTGTGTGTG | 94°C – 3 minutes;  94°C – 1 min, 67°C – 1 min and 72°C – 1 min (5 times);  94°C – 1 min, 65°C – 1 min and 72°C 1 – min (5 times);  94°C – 1 min, 63°C – 1 min and 72°C – 1 min (5 times);  94°C – 1 min, 61°C – 1 min and 72°C – 1 min (30 times);  72°C – 10 min. |
| **24 sα** | | | |
| First round  Second round | D75  D76 | GCAGATCTTGGTTGGCGTAG GGTTCTCTGTTGCCCCTTTT | 94°C – 3 minutes;  94°C – 30 sec, 64°C – 45 sec and 72°C – 1 min (3 times);  94°C – 30 sec, 62°C – 45 sec and 72°C – 1 min (3 times);  94°C – 30 sec, 60°C – 45 sec and 72°C – 1 min (3 times);  94°C – 30 sec, 58°C – 45 sec and 72°C – 1 min (35 times);  72°C – 10 min. |
|  | D71  D76 | AAGGTGCGTCGACAGTGTGG GGTTCTCTGTTGCCCCTTTT | 94°C – 3 minutes;  94°C – 30 sec, 60°C – 30 sec and 72°C – 30 sec (6 times);  94°C – 30 sec, 57°C – 30 sec and 72°C – 30 sec (6 times);  94°C – 30 sec, 55°C – 30 sec and 72°C – 30 sec (30 times);  72°C – 10 min. |
| **A10** | | | |
| First round  Second round | Pr1  P6 | CCGCTAAGCAGTTCTGTCCATA GTGATCGCAGGAAACGTGA | 94°C – 3 minutes;  94°C – 1 min, 65°C – 1 min and 72°C – 1 min (35 times);  72°C – 10 min. |
|  | Pr1  Pr3M | CCGCTAAGCAGTTCTGTCCATA CGTGGCATGGGGTAATAAAGCA | 94°C – 3 minutes;  94°C – 1 min, 65°C – 1 min and 72°C – 1 min (35 times);  72°C – 10 min. |
