# Supplementary material for: Trypanosoma cruzi DTU parasite diversity and clinical outcomes in mesoregions of the Northeast Brazilian State of Pernambuco
Source: PLoS Negl Trop Dis. 2026 Feb 13;20(2):e0013996. doi: 10.1371/journal.pntd.0013996 (PMC12923128; doi:10.1371/journal.pntd.0013996)
Supplement: S2 Table — (DOCX) [file pntd.0013996.s002.docx]

**S2 Table. Concentrations and volumes used in PCR reactions for genotypic characterization of *Trypanosoma cruzi*.**

| **Reagents** | **Initial** | **Final** | **Volume for 1 reaction (µL)** |
| --- | --- | --- | --- |
| kDNA  Platinum Taq polymerase buffer  MgCl_2_  DNTPs (dATP, dCTP, dDTTP and dGTP)  121  122  Platinum Taq Enzyme  H_2_O  **Reaction mix volume**  DNA samples  Final volume | -  -  -  -  -  -  -  -  -  - | 10 x  25 mM  10 mM  100 ng/µL  100 ng/µL  5 U/mL  -  -  -  - | 5  8  1 each  1.5  1.5  0.25  24.74  **45**  5  50 |
| β-globin  Platinum Taq polymerase buffer  MgCl_2_  DNTPs (dATP, dCTP, dDTTP and dGTP)  PCO3  PCO4  Platinum Taq Enzyme  H_2_O  **Reaction mix volume**  DNA samples  Final volume | -  -  -  -  -  -  -  -  -  - | 10 x  25 mM  10 mM  100 ng/µL  100 ng/µL  5 U/mL  -  -  -  - | 5  8  1 each  1.5  1.5  0.25  24.74  **45**  5  50 |
| SL-IRac  GoTaq Green Master Mix  UTCC  TCac  H_2_O  **Reaction mix volume**  DNA samples  Final volume | 2 x  50 µM  50 µM  -  -  -  - | 1 x  1.7 µM  1.7 µM  -  -  -  - | 15  1  1  8  **25**  5  30 |
| SL-IR I and II  GoTaq Green Master Mix  TCC  TC1  TC2  H_2_O  **Reaction mix volume**  DNA samples  Final volume | 2 x  50 µM  50 µM  50 µM  -  -  -  - | 1 x  0.5 µM  0.5 µM  0.5 µM  -  -  - | 15  0.3  0.3  0.3  9.1  **25**  5  30 |
| 24Sα rDNAfirst round (heminested PCR)  GoTaq Green Master Mix  D75  D76  H_2_O  **Reaction mix volume**  DNA samples  Final volume | 2 x  50 µM  50 µM  -  -  -  - | 1 x  1 µM  1 µM  -  -  -  - | 15  0.6  0.6  8.8  **25**  5  30 |
| 24Sα rDNAfirst round (heminested PCR)  GoTaq Green Master Mix  D71  D76  H_2_O  **Reaction mix volume**  PCR product (from the first round)  Final volume | 2 x  50 µM  50 µM  -  -  -  - | 1 x  1 µM  1 µM  -  -  -  - | 15  0.6  0.6  12.8  **29**  1  30 |
| A10 first round (heminested PCR)  GoTaq Green Master Mix  Pr1  P6  H_2_O  **Reaction mix volume**  DNA samples  Final volume | 2 x  50 µM  50 µM  -  -  -  - | 1 x  0.6 µM  0.6 µM  -  -  -  - | 15  0.36  0.36  9.28  **25**  5  30 |
| 24Sα rDNAfirst round (heminested PCR)  GoTaq Green Master Mix  Pr1  Pr3  H_2_O  **Reaction mix volume**  PCR product (from the first round)  Final volume | 2 x  50 µM  50 µM  -  -  -  - | 1 x  0.6 µM  0.6 µM  -  -  -  - | 15  0.36  0.36  13.28  **29**  1  30 |
