# Supplementary material for: Trypanosoma cruzi DTU parasite diversity and clinical outcomes in mesoregions of the Northeast Brazilian State of Pernambuco
Source: PLoS Negl Trop Dis. 2026 Feb 13;20(2):e0013996. doi: 10.1371/journal.pntd.0013996 (PMC12923128; doi:10.1371/journal.pntd.0013996)
Supplement: S3 Table — (DOCX) [file pntd.0013996.s003.docx]

**S3 Table. Data of non-etiologically treated patient samples positive for *Trypanosoma cruzi* kDNA and genotyped for DTU distributed according to birthplace (Pernambuco, Brazil) and clinical form.**

| **Patient** | **Gender** | **Age** | **Birthplace (city)** | **Mesoregion** | **Clincal form** | **Target genes** | | | | **DTU** |
| --- | --- | --- | --- | --- | --- | --- | --- | --- | --- | --- |
|  | | | | | | **SL-IRac** | **SL-IR I and II** | **24sα-rDNA** | **A10** |  |
| DPE 2 | M | 69 | Vitória de Santo Antão | Mata | DIG | Neg | 300bp | 125bp | Neg | TcV |
| DPE 6 | M | 35 | Pesqueira | Agreste | CARD | Neg | 300bp | 140bp | Neg | TcII/TcVI |
| DPE 7 | M | 73 | Recife | RM | CARD | Neg | 300bp | 125bp | Neg | TcV |
| DPE 10 | F | 53 | Orobó | Agreste | CARD | 200 | Neg | 140bp | Neg | TcIV |
| DPE 15 | M | 81 | Lagoa do Carro | Mata | CARD | Neg | 300bp+350bp | 125bp | Neg | TcI+TcV |
| DPE 19 | M | 75 | Carnaíba | Sertão | CARD | 200bp | Neg | 140bp | Neg | TcIV |
| DPE 22 | F | 56 | Vicencia | Mata | DIG | Neg | 300bp | Neg | Neg | TcII/TcV/TcVI |
| DPE 28 | F | 48 | Afogados da Ingazeira | Sertão | CARD/DIG | Neg | 300bp | Neg | Neg | TcII/TcV/TcVI |
| DPE 32 | F | 78 | Nazaré da Mata | Mata | CARD | Neg | 300bp | 125bp/140bp | Neg | TcV+TcII/TcVI |
| DPE 34 | M | 73 | Nazaré da Mata | Mata | CARD/DIG | Neg | 300bp | Neg | Neg | TcII/TcV/TcVI |
| DPE 36 | M | 56 | São Vicente Ferrer | Agreste | CARD | 200bp | Neg | Neg | Neg | TcIII/TcIV |
| DPE 39 | F | 50 | Água Preta | Mata | CARD | 200bp | Neg | 125bp | Neg | TcIII |
| DPE 43 | M | 39 | Jaboatão dos Guararapes | RM | Non-CARD | 200bp | Neg | 140bp | Neg | TcIV |
| DPE 46 | F | 59 | Limoeiro | Agreste | CARD | Neg | 300bp | 125bp | Neg | TcV |
| DPE 50 | M | 60 | Feira Nova | Agreste | CARD | 200bp | Neg | 125bp | Neg | TcIII |
| DPE 51 | F | 50 | Buique | Agreste | Non-CARD | Neg | 300bp | 125bp | Neg | TcV |
| DPE 54 | M | 66 | Afogados da Ingazeira | Sertão | CARD | Neg | 300bp | 125bp | Neg | TcV |
| DPE 55 | M | 68 | Salgueiro | Sertão | CARD | 200bp | Neg | Neg | Neg | TcIII/TcIV |
| DPE 56 | F | 57 | Vicencia | Mata | CARD | 200bp | Neg | 140bp | Neg | TcIV |
| DPE 63 | M | 63 | Buique | Agreste | CARD | Neg | 300bp | 140bp | Neg | TcII/TcVI |
| DPE 69 | F | 60 | Bom Jardim | Agreste | CARD | 200bp | Neg | 125bp | Neg | TcIII |
| DPE 70 | F | 65 | Recife | RM | CARD | Neg | 300bp | Neg | Neg | TcII/TcV/TcVI |
| DPE 73 | F | 76 | Vicencia | Mata | CARD | 200bp | Neg | 125bp+140bp | Neg | TcIII+TcIV |
| DPE 76 | F | 67 | Vicencia | Mata | CARD/DIG | 200bp | Neg | 125bp | Neg | TcIII |
| DPE 78 | F | 54 | Timbaúba | Mata | CARD | Neg | 300bp | 125bp | Neg | TcV |
| DPE 84 | F | 70 | Timbaúba | Mata | CARD | 200bp | Neg | 125bp | Neg | TcIII |
| DPE 86 | M | 60 | Carnaíba | Sertão | Non-CARD | 200bp | Neg | Neg | Neg | TcIII/TcIV |
| DPE 88 | F | 55 | Itapetim | Sertão | CARD | 200bp | Neg | 125bp | Neg | TcIII |
| DPE 89 | F | 53 | Bom Jardim | Agreste | CARD/DIG | 200bp | Neg | 140bp | Neg | TcIV |
| DPE 90 | F | 61 | Nazaré da Mata | Mata | CARD | Neg | 300bp | 140bp | Neg | TcII/TcVI |
| DPE 94 | F | 68 | Pau Dalho | Mata | CARD | Neg | 300bp+350bp | 125bp | Neg | TcI+TcV |
| DPE 98 | M | 53 | São Joaquim do Monte | Agreste | Non-CARD | Neg | 300bp | 140bp | Neg | TcII/TcVI |
| DPE 100 | M | 62 | Buique | Agreste | Non-CARD | Neg | 300bp | 140bp | 525bp | TcVI |
| DPE 104 | F | 36 | Afogados da Ingazeira | Sertão | CARD | Neg | 300bp | 125bp | Neg | TcV |
| DPE 106 | M | 55 | Buique | Agreste | CARD | Neg | 350bp | Neg | Neg | TcI |
| DPE 119 | F | 49 | Machado | Agreste | CARD | Neg | 300bp | 125bp | Neg | TcV |
| DPE 122 | F | 48 | Carnaubeira da Penha | Sertão | CARD/DIG | 200bp | Neg | 125bp | Neg | TcIII |
| DPE 129 | M | 60 | Altinho | Agreste | CARD | Neg | 300bp | 140bp | 580bp | TcII |
| DPE 131 | F | 72 | Pau Dalho | Mata | CARD | 200bp+157bp | 300bp | 125bp | Neg | TcIII+TcV |
| DPE 133 | F | 62 | Carpina | Mata | CARD | 200bp | Neg | 125bp | Neg | TcIII |
| DPE 134 | F | 60 | São Lourenço da Mata | RM | CARD | Neg | 300bp | 125bp | Neg | TcV |
| DPE 144 | M | 30 | Recife | RM | CARD | Neg | 300bp | 140bp | 525bp | TcVI |
| DPE 145 | F | 55 | Flores | Sertão | CARD | 200bp | Neg | 125bp | Neg | TcIII |
| DPE 146 | F | 66 | Timbaúba | Mata | CARD/DIG | Neg | 300bp | 140bp | 525bp | TcVI |
| DPE 148 | M | 69 | Vicencia | Mata | CARD | 157bp | 300bp | 125bp | Neg | TcV |
| DPE 153 | F | 50 | Timbaúba | Mata | CARD | Neg | 300bp | 125bp | Neg | TcV |
| DPE 154 | M | 77 | Vicencia | Mata | CARD/DIG | 200bp | Neg | 140bp | Neg | TcIV |
| DPE 155 | F | 73 | Macaparana | Mata | CARD/DIG | Neg | 300bp | 140bp | Neg | TcII/TcVI |
| DPE 158 | F | 62 | Machados | Agreste | CARD | 200bp | Neg | 125bp | Neg | TcIII |
| DPE 159 | F | 42 | Vicencia | Mata | CARD | Neg | 300bp | 125bp | Neg | TcV |
| DPE 165 | F | 76 | Recife | RM | CARD/DIG | Neg | 350bp | Neg | Neg | TcI |
| DPE 166 | F | 70 | Vicencia | Mata | CARD/DIG | Neg | 300bp | Neg | Neg | TcII/TcV/TcVI |
| DPE 167 | F | 41 | Recife | RM | CARD | Neg | 300bp | Neg | Neg | TcIII/TcIV |
| DPE 171 | M | 18 | Limoeiro | Agreste | Non-CARD | Neg | 300bp | 140bp | Neg | TcII/TcVI |
| DPE 202 | F | 84 | Bom Jardim | Agreste | DIG | Neg | 300bp | Neg | Neg | TcII/TcV/TcVI |
| DPE 204 | F | 65 | Nazaré da Mata | Mata | CARD | Neg | 300bp | 125bp | Neg | TcV |
| DPE 215 | M | 71 | São Vicente Ferrer | Agreste | CARD | Neg | 300bp | Neg | Neg | TcII/TcV/TcVI |
| DPE 216 | F | 72 | Vicencia | Mata | CARD | 150bp | 300bp+350bp | Neg | Neg | TcI+TcII/TcV/TcVI |
| DPE 219 | F | 70 | Salgueiro | Sertão | CARD | Neg | 300bp+350bp | 140bp | Neg | TcI+TcII/TcVI |
| DPE 225 | F | 58 | Vicencia | Mata | CARD | Neg | 300bp | Neg | Neg | TcII/TcV/TcVI |
| DPE 226 | F | 64 | Timbaúba | Mata | CARD | 200bp | Neg | 125bp | Neg | TcIII |
| DPE 228 | F | 40 | Timbaúba | Mata | CARD | 200bp | Neg | Neg | Neg | TcIII/TcIV |
| DPE 229 | M | 60 | Bonito | Agreste | CARD | 200bp | Neg | 125bp | Neg | TcIII |
| DPE 230 | M | 35 | Sertania | Sertão | CARD | 200bp | Neg | 125bp+140bp | Neg | TcIII+TcIV |
| DPE 232 | F | 62 | São Lourenço da Mata | RM | CARD/DIG | 200bp | Neg | 140bp | Neg | TcIV |
| DPE 233 | M | 77 | Gloria do Goita | Mata | CARD | Neg | 300bp | Neg | Neg | TcII/TcV/TcVI |
| DPE 234 | F | 59 | Bom Jardim | Agreste | CARD | 200bp | Neg | 125bp+140bp | Neg | TcIII+TcIV |
| DPE 235 | F | 63 | Timbaúba | Mata | CARD | Neg | 300bp | Neg | Neg | TcII/TcV/TcVI |
| DPE 239 | F | 54 | Catende | Mata | CARD | Neg | 300bp | Neg | Neg | TcII/TcV/TcVI |
| DPE 247 | M | 60 | Vicencia | Mata | CARD | 157bp | 300bp+350bp | Neg | Neg | TcI+TcII/TcV/TcVI |
| DPE 251 | M | 69 | Pau Dalho | Mata | CARD | 157bp | Neg | Neg | Neg | TcII/TcV/TcVI |
| DPE 254 | F | 68 | Aliança | Mata | CARD | 200bp | Neg | Neg | Neg | TcIII/TcIV |
| DPE 257 | F | 72 | Timbaúba | Mata | CARD | 200bp | Neg | Neg | Neg | TcIII/TcIV |
| DPE 273 | M | 79 | Gloria do Goita | Mata | Non-CARD | 200bp | Neg | Neg | Neg | TcIII/TcIV |
| DPE 280 | F | 62 | Bom Jardim | Agreste | CARD | 200bp | Neg | Neg | Neg | TcIII/TcIV |
| DPE 282 | M | 57 | Carnaiba | Sertão | DIG | 200bp | Neg | Neg | Neg | TcIII/TcIV |
| DPE 287 | M | 65 | Quipapa | Mata | CARD/DIG | 200bp | Neg | 125bp | Neg | TcIII |
| DPE 291 | F | 60 | Panelas | Agreste | CARD | Neg | 300bp | 125bp | Neg | TcV |
| DPE 301 | F | 55 | Quipapa | Mata | CARD | 150bp/157bp | 350bp | Neg | Neg | TcI |
| DPE 304 | M | 73 | Aliança | Mata | DIG | Neg | 300bp | 125bp | Neg | TcV |
| DPE 305 | M | 70 | São Benedito do Sul | Mata | CARD | Neg | 300bp | 140bp | Neg | TcII/TcVI |
| DPE 312 | F | 70 | Limoeiro | Agreste | CARD | 150bp/157bp | 300bp | Neg | Neg | TcII/TcV/TcVI |
| DPE 319 | F | 49 | Sertania | Sertão | Non-CARD | 150bp/157bp+200bp | 300bp+350bp | Neg | Neg | TcI+TcII/TcV/TcVI+TcIII/TcIV |
| DPE 325 | M | 59 | Alagoinha | Agreste | Non-CARD | 200bp | Neg | Neg | Neg | TcIII/TcIV |
