# Supplementary figures and images for: Trypanosoma cruzi DTU parasite diversity and clinical outcomes in mesoregions of the Northeast Brazilian State of Pernambuco
Source: PLoS Negl Trop Dis. 2026 Feb 13;20(2):e0013996. doi: 10.1371/journal.pntd.0013996 (PMC12923128; doi:10.1371/journal.pntd.0013996)

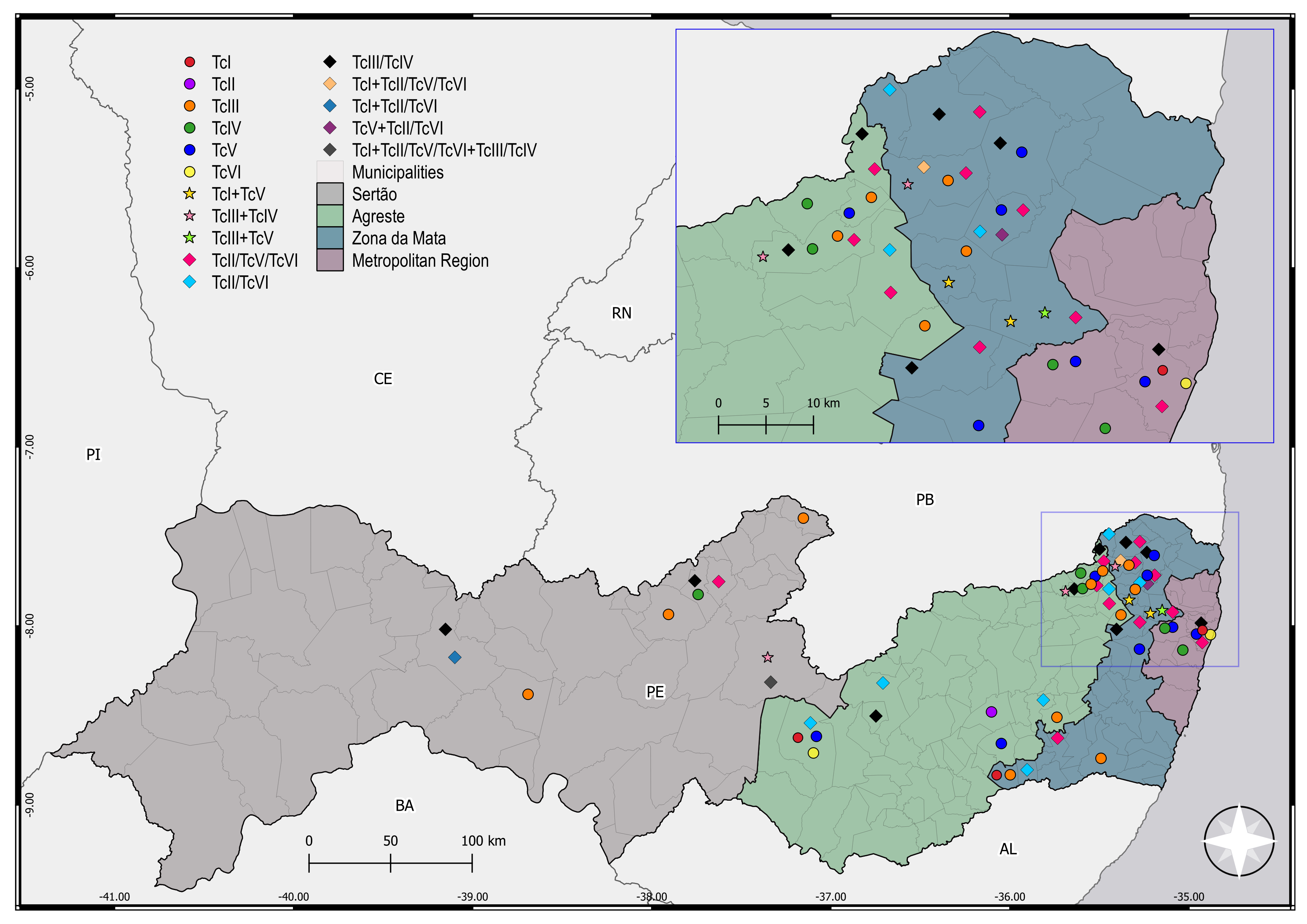

Supplement: S1 Fig — This map was created using QGIS 3.34 ‘Prizren’ version software and cartographic bases obtained from the Brazilian Institute of Geography and Statistics https://www.ibge.gov.br/geociencias/organizacao-do-territorio/malhas-territoriais/15774-malhas.html. (TIF) [file pntd.0013996.s004.tif]
